# Supplementary material for: Chronic Stress Segregates Mice into Distinct Behavioral Phenotypes Based on Glucocorticoid Sensitivity
Source: Int J Mol Sci. 2025 Nov 26;26(23):11436. doi: 10.3390/ijms262311436 (PMC12692131; doi:10.3390/ijms262311436)
Supplement: Supplementary file 1 [file ijms-26-11436-s001.zip › ijms-3957130-supplementary/FigureS1.pdf]

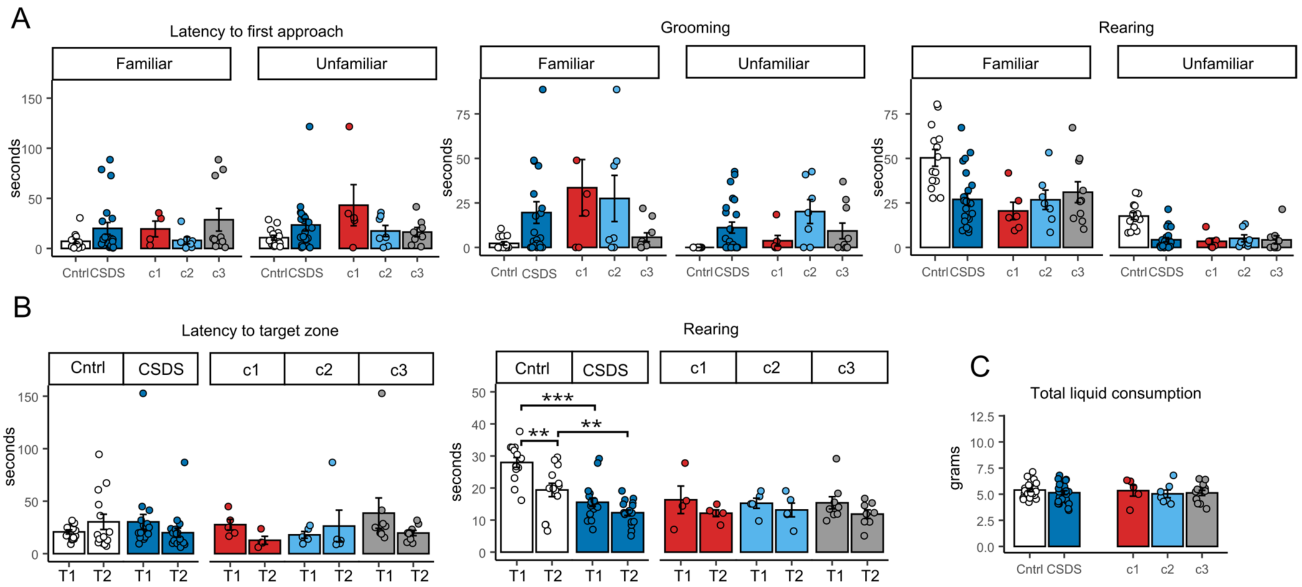

Figure S1. Characteristics of mice behavior after chronic social defeat stress exposure. A. Partition. Familiar partner - male CD1 with whom the experimental mice were kept in the home cage for the last day, unfamiliar - intact CD1 males. B. Social interaction test. In the first trial, testing was carried out without a social partner, in the second trial, CD1 aggressor was placed. t1 - trial 1, t2 - trial 2. C. Sucrose preference test. Clusters c1, c2 and c3 are CSDS subgroups identified based on k-means cluster analysis by response to dexamethasone administration . c1 (cluster 1): low basal corticosterone + abnormal response to dexamethasone (paradoxical increase). c2 (cluster 2): high basal corticosterone and blunted suppression after dexamethasone (≤2-fold decrease). c3 (cluster 3): low basal corticosterone and control-like suppression after dexamethasone. One- or two-way ANOVA, TukeyHSD, * p adj < 0.05, ** p adj < 0.01, *** p adj < 0.001.
